# The Effect of Lymphoedema Exercises and Foot Elevation on the Quality of Life of Patients with Elephantiasis

**DOI:** 10.1155/2020/6309630

**Published:** 2020-05-27

**Authors:** Emirensiana Watu, Supargiyono, Haryani

**Affiliations:** ^1^Magister of Nursing Student, Faculty of Medicine, Public Health and Nursing, Universitas Gadjah Mada, Yogyakarta, Indonesia; ^2^Department of Parasitology, Faculty of Medicine, Public Health and Nursing, Universitas Gadjah Mada, Yogyakarta, Indonesia; ^3^Department of Medical Surgical Nursing, Faculty of Medicine, Public Health and Nursing, Universitas Gadjah Mada, Yogyakarta, Indonesia

## Abstract

Filariasis is a chronic infectious disease caused by filarial worms. Swelling in the legs in patients with filariasis can result in a significantly lower quality of life. The recommended treatments for patients who experience swelling or lymphoedema are lymphoedema exercises and foot elevation. This research is a quantitative study with a quasi-experimental design including pre- and posttreatment tests with a control group. This study used a cluster sampling method, which is a nonprobability sampling technique. The samples in this study were 48 respondents divided into two groups: 24 respondents from the Nebe Village comprising the intervention group and 24 respondents from the Bangkoor Village comprising the control group. The intervention group conducted lymphoedema exercises and foot elevation three times a week for 15–20 min for 1 month and measured their quality of life using the LFSQQ questionnaire. Measurements of pitting edema and ankle diameter were also carried out. Paired *t*-test revealed an improvement in the quality of life between pretest and posttest in the intervention and control groups (*p*=0.001). The quality of life in the pre-post intervention group improved from 67.42 to 81.58. In addition, the quality of life in the pre-post control group only improved from 62.50 to 72.58. The level of pitting edema decreased from severe (+++) to moderate (++) and from mild (+) to normal (0), and there was no difference in ankle diameter in each group (*p*=1.000). The quality of life improved before and after the administration of lymphoedema exercises and foot elevation for each group. Pitting edema decreased before and after lymphoedema exercises and foot elevation for each group. There was no decrease in ankle diameter after lymphoedema exercises and foot elevation in the intervention and control groups.

## 1. Introduction

Filariasis is a chronic infectious disease caused by filarial worms. Three types of parasitic worms cause this infection: *Wuchereria bancrofti*, *Brugia malayi*, and *Brugia timori*. These parasites are transmitted by many species of mosquitoes including *Anopheles*, *Culex*, *Aedes*, and *Mansonia* [1]. The impact of this filariasis infection can occur acutely in the form of inflammation of glandular and lymphatic channels (adenolymphangitis) and chronic symptoms due to blockage of lymph flow, which can cause enlarged legs resembling an elephant's legs. Therefore, the disease is known as elephantiasis. Sufferers of filariasis experience functional disorders, emotional distress, and poor quality of life [2]. Globally, an estimated 120 million people in the tropics and subtropics are infected with lymphatic filariasis. Around 856 million people in the world are at risk for filariasis, and around 40 million people are disabled and paralyzed by the disease. On the basis of data reported by the Provincial Health Office and the results of a survey in Indonesia, among 34 provinces in Indonesia, filariasis was found in three provinces: East Nusa Tenggara, Aceh, and West Papua Provinces. The total number of chronic cases of filariasis in Indonesia increased every year from 2002 to 2014. Between 2014 and 2015, filariasis cases in Indonesia decreased from 14,392 to 13,032 [3]. Filariasis cases in Sikka District in 2016 increased to 356, with most clinical filariasis cases found in Watubaing health center [4]. Lymphoedema in patients with filariasis has an extremely negative impact on their quality of life. This decrease in quality of life can lead to physical, functional, and emotional disorders; economic burden; loss of mobility; and psychosocial burden [5]. In addition to the physical disabilities associated with lymphoedema and hydrocele, sufferers often experience social rejection, stigma, and discrimination. These adverse consequences have a large impact on the quality of life and functioning of sufferers. Therefore, the disease needs close medical attention, proper management [6], and morbidity control [6]. Lymphoedema exercises and foot elevation administered over a long period can alleviate swollen limbs and prevent additional swelling. The therapeutic function of this exercise is to train the leg muscles that act as a pump to alleviate fluid build-up and prevent joint hardening [7].

## 2. Methods

### 2.1. Settings and Participants

This research was conducted after obtaining permission from the ethical clearance committee of the Universitas Gadjah Mada in Yogyakarta on April 16, 2019, with No. KE/FK/0419/EC/2019. This research is a quantitative study with a quasi-experimental design including pre- and posttreatment tests with a control group. This study used a cluster sampling method, which is a nonprobability sampling technique. The samples in this study were 48 participants divided into two groups: 24 participants from the Nebe Village comprised the intervention group and 24 participants from the Bangkoor Village comprised the control group. Participants were accepted based on the following inclusion criteria: participants had to be experiencing elephantiasis, aged 18 years or older, exhibit lymphoedema of degree of 1–3, and willing to become participants. Exclusion criteria were as follows: patients suffering from primary lymphoedema (lymphatic system disorders and others), participants with severe psychological disorders, and participants with lymphoedema grade 4 to degree 7. Participants were declared dropped if they did not follow the research procedures properly. The intervention group did lymphoedema exercises and foot elevation for 15–20  min three times per week for 1 month. The control group was treated with foot elevation only at the first meeting in the first week and the last meeting in the fourth week. Foot elevation in the control group was conducted by each participant at their own convenient time without supervision by the researcher. The participant recruitment process was determined by the researchers working in locations in the two villages. Some participants were dropped because they could not follow the procedure several times; some were dropped because they failed to complete the lymphoedema exercises regularly, and a few participants moved their residence and could not be tracked.

### 2.2. Intervention

Lymphoedema exercises and foot elevation were administered to the intervention group, while the control group was only treated with foot elevation. The researchers took pretest measurements for both groups with a quality of life questionnaire LFSQQ (Lymphatic Filariasis Specific Quality of Life Questionnaire) with a score of 0–100, in which 100 is the highest quality of life [8, 9], and demographic data questionnaires. In addition, pitting edema and ankle diameter were measured for both groups using a tape measure. Treatment was given to respondents in sessions around 20 min. In the intervention group, lymphoedema exercises and elevation were conducted at home with the monitoring process. Monitoring was conducted every week. The control group was given foot elevation instructions provided by using a leaflet. This exercise was also carried out at home. For the control group, there was no weekly monitoring, and weekly elevation was done at any time according to the patient's own discretion. Lymphoedema exercises and elevation in the intervention group were conducted three times a week on a schedule set by each respondent for himself/herself.

### 2.3. Outcome Measurement

Data were collected by meeting directly with respondents and distributing demographic questionnaires and LFSQQ (Lymphatic Filariasis Specific Quality of Life Questionnaire). The LFSQQ were previously tested for validity and reliability with 35 respondents from Puskesmas Tana Rawa who experienced elephantiasis. This validity test obtained a score (*r* = 0.346–0.802). Cronbach's alpha was *α* = 0.94. In addition, measurements of pitting edema and ankle diameter measurements were obtained using a measuring tape.

## 3. Statistics

The normality distribution of the data was tested using the Shapiro–Wilk test because of the small sample size (*n* < 50). In this study, a paired *t*-test was conducted if the spread of the data was normal. If the spread distribution was not normal, then the Wilcoxon test was used. To compare the intervention group and the control group, an independent *t*-test was conducted to determine the difference in the average between the two groups. If the data were not normally distributed, then the Mann–Whitney *U* test was performed.

## 4. Results

### 4.1. Characteristics of Respondents

Univariate analysis was performed to describe the characteristics of the participants. Characteristics included age, sex, marital status, highest level of education achieved, occupation, income, and degree of lymphoedema. All respondents' characteristics are homogeneous with *p* > 0.05. The characteristics of the respondents are presented in Table 1.

Table 1 shows that the demographic characteristics of age, sex, occupation, last education, income, degree of lymphoedema, and length of suffering from elephantiasis in all groups statistically presented homogeneous characteristics (*p* > 0.05). Thus, there was no significant difference between the characteristics of the participants in the intervention group and in the control group. A majority of respondents (37.5%) were in the old age category in the intervention group and late elderly (41.7%) in the control group. Participants in this study were dominated by women in the intervention group (83.3%) and in the control group (75%). The majority of the intervention and control groups worked as farmers. In addition, most respondents reported the last education at the elementary school level and incomes below <1,000,000 IDR/month. The majority of lymphoedema sufferers in the intervention group exhibited symptoms of grade I (41.7%), whereas 50% of respondents in the control group exhibited stage I symptoms. Approximately 37.5% of intervention respondents experienced swelling in the legs for more than 11–12 years, whereas 83.3% in the control group experienced swelling for less than 10 years.

### 4.2. Quality of Life in the Intervention and Control Groups

In Table 2, the results of the pre-post-test analysis in the intervention group and the control group show the value of *p*=0.001 (*p* < 0.05). Therefore, the quality of life in the both groups was improved which were before and after lymphoedema exercises and foot elevation in the intervention group and foot elevation only in the control group.

### 4.3. Pitting Edema in the Intervention and Control Groups

As shown in Table 3, in the intervention group after the intervention, the number of participants with normal pitting edema (0) increased by four people, with mild pitting edema (+) decreased by four people, with moderate pitting edema (++) increased by one person, and severe (+++) decreased by one person. By contrast, in the control group after being given treatment, respondents with normal pitting edema (0) increased by two people, mild (+) increased by 13 people, moderate (++) decreased by one person, and severe (+++) became absent.

### 4.4. Ankle Diameter in the Intervention and Control Groups

Table 4 shows no significant decrease in ankle diameter before and after lymphoedema exercises and foot elevation in the intervention group and foot elevation only in the control group (*p* > 0.05).

## 5. Discussion

### 5.1. Characteristics of Participant

The age demographic of participants was dominated by patients aged 56–65 years (late elderly) in the control group and 65 years and over (elderly) in the intervention group. Filariasis transmission can occur to anyone, regardless of age. Clinical manifestations of lymphatic filariasis have risen over the years, and this disease rarely displays obvious symptoms in children. Anyone can become infected with filariasis if they are bitten by an infected mosquito containing stage three larvae. In endemic areas of filariasis, not all people are infected, and even infected people do not always show clinical symptoms. Despite the absence of clinical symptoms, pathological changes occur in the body [10]. Elephantiasis is more common in women than in men, which was reflected in the demographic data of both the intervention and control groups. In this study [11], the physical differences between men and women led to the risk of infection and disease manifestations. In addition, women's socioeconomic activities inside and outside the home can intensify the symptoms. However, according to research conducted in India, the development of lymphoedema across men and women displays similar proportionality. The results of this test showed that most respondents work as farmers in the intervention and control groups. Workers in the field of agriculture display a heightened incidence of lymphatic filariasis. This research was in line with a previous study [10], which explained that working as a farmer requires routine activities that can heighten exposure of an individual or a community to mosquito-borne illnesses. Farmers must work frequently in fields, rice fields, or forests, which are breeding grounds for mosquitoes [12]. In addition, farmers do not always wear footwear or protective long pants, so the risk of contracting filariasis is high. Another postulate links filariasis with outdoor occupations in cold weather, which can intensify elephantiasis symptoms [13]. The average education level achieved in both the intervention and control groups was relatively low at around the elementary level. In theory, more educated individuals may have a better knowledge of disease progression and be more likely to seek medical attention to inhibit the disease than individuals with low education levels. The average monthly income for the two groups was <Rp.1,000,000. According to research from [14], the level of income implies the economic capacity for individuals to meet decent food and clothing standards. This includes having permanent housing without gaps or holes in the walls, which can provide opportunities for the entry of mosquitoes. High-income individuals may also have access to better health services than low-income individuals. The data indicated that a majority of respondents in the intervention and control groups had elephantiasis symptoms or swollen feet of stage I. In stage I lymphoedema, swelling in the legs disappears when you wake up in the morning, and there are no symptoms of skin folds, nodules, mossy lesions, and no obstruction due to the large foot size [15]. The average respondent experienced this disease for 11–12 years both in the intervention and in control groups. Symptoms of foot swelling arise due to a blockage in the lymph vessels that usually occurs. This is caused by the macrofilaria parasite, which survives for a long time in the lymph glands. Damage can occur to the lymph system due to the breeding of worms. The microfilaria parasite usually dies by itself after 5–7 years, but some severe lymphatic system damage may be irreversible [15].

### 5.2. Effects of Administration of Lymphoedema and Foot Elevation on Quality of Life

The results of the pre- and poststudies in the intervention group were *p*=0.000(*p* < 0.05). Also, in the pre-post control group, the results were *p*=0.000(*p* < 0.05). These values indicate an increase in quality of life before and after the lymphoedema intervention and foot elevation in the intervention and control groups. According to [5], the decrease in quality of life in patients who experience lymphoedema can be influenced by acute adenolymphangitis attacks that occur in patients. This can cause pain and discomfort, rest and sleep disturbance, disruption of daily activities, dependence on treatment and care, and reduction of work capacity and necessitate social support. In addition, a previous study [16] explained that many patients with lymphatic filariasis experience social, psychological, and physical disabilities. This underpins the importance of morbidity control in patients who have been exposed to filarial lymphoedema. Morbidity management programs must be expanded to include provisions for counseling, rehabilitation, and health education. Management of psychosocial problems caused by lymphatic filariasis should also involve simple and effective hygiene measures, including frequent washing of feet with soap and water and regular limb exercises [16]. Damage to the lymphatic system is permanent, so the administration of antifilaria drugs will not cure lymphoedema. However, lymphoedema can be managed so that acute attacks stop and the disease does not worsen. Without proper management, lymphoedema sufferers will have progressive problems that can worsen their socioeconomic situation; patients may have difficulty to work or go on to further education. Sufferers often feel alone and sometimes lose friends, family, or work. However, proper management of lymphoedema can provide sufferers with new hope for a better life. This study failed to demonstrate a significant difference between the intervention group and the control group in that both groups presented an indistinguishably improved quality of life before and after the intervention of lymphoedema exercises and foot elevation (*p*=0.000; *p* < 0.05). A previous study [17] explained that lymphoedema exercises and foot elevation are some of many exercises designed to involve repetitive movements that activate a musculoskeletal response and provide the additional benefit of compression. These exercises can improve lymphatic flow and increase lymphatic protein absorption. In addition to lymphoedema exercises, management in the form of foot elevation is useful to reduce the accumulation of lymphatic fluid, which can move along the vertical axis proximally and distally. Leg elevation is not effective at the stage of severe lymphoedema, especially in cases of extensive and severe fibrosclerosis. Thus, the two methods can provide the same benefits in improving the quality of life. Management of lymphoedema caused by lymphatic filariasis, such as performing skin care routines and simple exercises, is an effective method of intervention to improve the quality of life of sufferers. In a study conducted by [18], the prevention of filariasis is crucial in preventing the occurrence of disabilities. Rehabilitation to enable sufferers of filariasis to maintain their daily lives includes physical, psychological, social, and economic aspects. Effective treatment is needed to reduce physical complaints and rehabilitative management.

### 5.3. Effects of Administration of Lymphoedema Exercises and Foot Elevation on Changes in Pitting Edema

In this study, the level of pitting edema decreased from severe (+++) to moderate (++) and from mild (+) to normal (0) in both the control and intervention groups. The normal lymphatic system can handle the amount of water protein without edema. Lymphoedema exhibits an imbalance in the lymphatic burden and lymphatic transport capability. Failure of lymph node output occurs in lymphatic drainage disorders. An imbalance of lymphatic fluid formation and lymphatic fluid absorption results in edema. Failure to control the swelling can cause recurring infections, progressive skin changes (elephantiasis), and external deformity. In most patients with elephantiasis, limb swelling occurs unilaterally or sometimes bilaterally but usually asymmetrically. This condition causes long-lasting changes and is associated with thickening of the skin, recurring fever, and pain in the affected parts [19]. A study conducted by [10] found that skin thickening and hardening will occur in patients who experience elephantiasis, as well as hyperpigmentation, hyperkeratosis, and an increase in the formation of connective tissue (fibrosis tissue formation). These effects are caused by repeated bacterial and fungal attacks, resulting in increased swelling. Infection occurs, disappears, and reoccurs until the swelling finally becomes permanent. The chronic phase of patients with elephantiasis with stage I symptoms is characterized by swelling in the body members and swollen skin, which will continue to experience pitting edema after being pressed for a few seconds. In stage II, symptoms of swelling do not disappear, and pitting edema begins to occur. Stage III is marked by persistent swelling and no swelling of pitting edema [19].

### 5.4. Effects of Administration of Lymphoedema and Foot Elevation on Changes in Ankle Diameter

From the results of the study described above, no difference was found in ankle diameter in the intervention and control groups before and after the administration of lymphoedema exercises and foot elevation (*p*=1.000; *p* > 0.05). The results of the analysis of the measurement of the diameter of the legs between the intervention group and the control group demonstrated a *p* value of 0.716 (*p* > 0.05). Thus, the researchers found no significant differences, perhaps due to the short time given for the study.

To achieve changes in foot diameter, therapeutic exercises must be conducted for more than 3 months. This is evidenced by research conducted by [20], which studied patients with neglected filariasis in India. Help was extended by providing education and physical training to reduce the swelling of the legs and thighs. This intervention was carried out in southern Indian regions (districts), specifically in Gulbarga in Karnataka (GK) and Alleppey in Kerala (AK). The sample size was 730 patients. In less than 3.5 months, they noted significant changes in the circumference of the legs and thighs. Efforts to continue to reduce lymphoedema should be continued every day, within dependent care and integrative care. This study also suggests that the technique patients use to wash their feet or compress their limbs should be closely examined. Medical professionals should also pay attention to the mentality of patients, who may feel inferior because of their condition. The study found that advertisement effects can be mitigated by practicing meditation or yoga [20]. The successful reduction of lymphoedema takes approximately 6 months. Additionally, one study [21] conducted in 27 patients found that the average volume of lymphoedema decreased significantly after 1 year. Leg volume tends to increase frequently with the onset of higher lymphoedema stages, so this trend also applies to lymphoedema affecting the foot. The feet are the most common site of lymphoedema in the endemic area of filariasis. The provision of lymphoedema exercises and foot elevation is crucial for patients who experience lymphoedema of the legs.

Such measures aim to help prevent fluid from collecting in the legs. Doing leg movements and elevation in the right position is important, especially on big and heavy feet. This process will presumably display significant results if carried out over a long period (1 year) with routine intensity.

In this study, basic lymphoedema management that emphasized exercise, hygiene, and self-care succeeded in reducing foot diameter. The use of compression bandages was found to be less effective than leg movements and elevation. Therefore, the management of basic lymphoedema is quite feasible and effective, and it can be applied in remote and limited human resource areas for the treatment and reduction of leg swelling due to lymphatic filariasis.

## 6. Conclusion

Provision of lymphoedema exercises and elevation can improve the quality of life of patients with elephantiasis and reduce pitting edema but does not decrease the ankle diameter of patients with elephantiasis.

## Figures and Tables

**Table 1:** Characteristics of the participants in Watubaing Health Center, Nebe Village and Bangkoor Village, April-May 2019 (*n* = 48).

Characteristics	Group				*p* value
	Intervention		Control		
	*n*	%	*n*	%	
*Age*					
Late adulthood (36–45 years)	3	12.5	0	0	0.96
Early elderly (46–55 years)	5	20.8	6	25	
Late elderly (56–65 years)	7	29.2	10	41.7	
Old age (>65 years)	9	37.5	8	33.3	
Mean ± SD	61.71 ± 13.063		61.08 ± 8.880		
*Gender*					
Male	4	16.7	6	25.0	1.000
Female	20	83.3	18	75.0	
*Occupation*					
Does not work	0	0	0	0	0.323
Civil servants	0	0	1	4.2	
Private	0	0	0	0	
IRT	0	0	0	0	
Farmers	24	100	23	95.8	
*Last education*					
Elementary school	24	100	23		0.323
Middle school	0	0	0		
High school	0	0	0		
S1	0	0	1		
*Income*					
<1,000,000	24	100	23	95.8	0.323
>1,000,000	0	0	1	4.2	
*Lymphoedema degree*					
First degree	10	41.7	12	50.0	0.757
Degree II	6	25.0	7	29.2	
Degrees III	8	33.3	5	20.8	
*Duration of elephantiasis disease*					
5–10 years	9	37.5	4	16.7	0.130
11–20 years	9	37.5	20	83.3	
>20 years	6	25	0	0	
Mean ± SD	14.71 ± 3.827		13.54 ± 2.797		

**Table 2:** Quality of life score of participants in the intervention group in Nebe Village and the control group in Bangkoor Village in the working area of Watubaing Maumere Health Center, 2019 (*n* = 48).

Quality of life		Group				
		Mean (SD)	Difference (SD)	Difference	CI 95%	*p* value
Intervention	Pretest	67.42 ± 6.121	14.16 (4.46)	0.48	(1.973–6.193)	0.001
	Posttest	81.58 ± 4.643				
Control	Pretest	62.50 ± 3.912	10.08 (2.44)			
	Posttest	72.58 ± 2.948				

**Table 3:** Pitting edema level of patients with elephantiasis in the intervention group in Nebe Village and the control group in Bangkoor Village in the working area of Watubaing Maumere Health Center, 2019 (*n* = 24).

	Before intervention				After intervention			
	(0)	(+)	(++)	(+++)	(0)	(+)	(++)	(+++)
	Normal	Mild	Moderate	Severe	Normal	Mild	Moderate	Severe
	*n* (%)	*n* (%)	*n* (%)	*n* (%)	*n* (%)	*n* (%)	*n* (%)	*n* (%)
Intervention group	6	14	2	2	10	10	3	1
	(25.0)	(58.3)	(8.3)	(8.3)	(41.7)	(41.7)	(12.5)	(4.2)
Control group	6	13	4	1	8	13	3	0
	(25.0)	(54.2)	(16.7)	(4.2)	(33.3)	(54.2)	(12.5)	(0)

**Table 4:** Results of the ankle diameter test for participants in the intervention group in Nebe Village and the control group in Bangkoor Village in the working area of Watubaing Maumere Health Center, 2019 (*n* = 48).

Ankle diameter	Median		*p* value Mann–Whitney
	(Minimum-maximum)		
	Intervention	Control	
Pretest	23.00 (0–38)	23.00 (0–38)	0.716
Posttest	23.00 (0–37)	23.00 (0–37)	
*p* value Wilcoxon test	1.000	1.000	

## Data Availability

No data were used to support this study.
